# Clinical evaluation of a new technique for custom-made spacers in septic two-stage revision of total hip arthroplasties

**DOI:** 10.1007/s00402-022-04748-z

**Published:** 2023-01-05

**Authors:** Moritz Mederake, Ulf Krister Hofmann, Bernd Fink

**Affiliations:** 1 Department of Trauma and Reconstructive Surgery, BG Klinik, University of Tübingen, Schnarrenbergstraße 95, 72076 Tübingen, Germany; 2 Department of Orthopaedic Surgery, University Hospital Tübingen, Hoppe Seyler-Str. 3, 72076 Tübingen, Germany; 3 Department of Orthopedic Trauma and Reconstructive Surgery, University of Aachen Medical Center, Pauwelsstraße 30, 52074 Aachen, Germany; 4 Department of Arthroplasty and Revision Arthroplasty, Orthopaedic Clinic Markgröningen GmbH, Kurt-Lindemann-Weg 10, 71706 Markgröningen, Germany; 5 Orthopaedic Department, University-Hospital Hamburg-Eppendorf, Martinistrasse 52, 20251 Hamburg, Germany

**Keywords:** Spacer, Periprosthetic joint infection, Hip arthroplasty, Two-stage revision, Antibiotic therapy, Orthopedic infections, Bone and joint infections

## Abstract

**Introduction:**

In septic two-stage revision surgery, success depends on numerous factors. Key steps are the procedure of ex- and reimplantation and the choice of spacer in the interim phase. The latter is still a matter of debate. Recently, we showed the microbial non-inferiority of a spacer technique using prosthetic cemented implants with an individualized antibiotic mixture in the cement applying a mechanically inferior cementation method. The aim of the present study was to evaluate the clinical results of these spacers in view of either an endofemoral or a transfemoral procedure.

**Materials and methods:**

Our collective consisted of 86 patients (45 endofemoral and 41 transfemoral procedures). The collective was analyzed with respect to complications, reinfection rate and clinical status at the end of the interim phase. Results of an endofemoral and transfemoral approach were compared.

**Results:**

With a median Staffelstein-Score of 60 (range 31–81) at the end of the interim phase, the first clinical results are promising. The reinfection-free rate after a median follow-up of 50 months was 90%. Spacer-related complications occurred in 8% of the total collective. Comparing the endo- and transfemoral procedure, there were no statistical differences in complications or regarding the clinical and infectiological outcome.

**Conclusions:**

In this study, we were able to show good clinical results for the presented spacer technique. With no relevant difference in outcome, the decision for an endofemoral or transfemoral technique can be based on technical deliberations. Further prospective comparative studies are necessary to show the clinical benefit of this procedure.

**Supplementary Information:**

The online version contains supplementary material available at 10.1007/s00402-022-04748-z.

## Introduction

Periprosthetic joint infections are serious complications of total hip arthroplasty and occur with an incidence of around 1–2%. In case of a late infection (later than 4 weeks after surgery), two therapeutic concepts are widely established: one- and two-stage septic revisions [8, 11, 19, 20].

The two-stage septic revision consists of two surgeries. In the first step, all foreign material is removed, radical debridement is performed, and either a spacer loaded with antibiotics is implanted or, alternatively, a sine–sine arthroplasty (Girdlestone situation) is created. In the second-stage operation, the spacer is removed and another radical debridement is performed, followed by the implantation of a new prosthesis. Between both steps, there is an interim phase of usually 6–12 weeks with targeted antibiotic therapy. After the implantation of the new prosthesis, the same protocol of antibiotic therapy as in the interim phase is administered for 6–12 weeks [11]. Two-stage revision concepts for infected hip endoprostheses have achieved success rates around 90% [5, 14, 18, 24].

The implanted spacer has several functions: to locally release antiinfective substances into the infected bed of the prosthesis and to minimize soft tissue contractions [5, 11]. There are several spacer techniques available: handformed or moulded techniques of pure cement spacers as well as prefabricated industrially produced antibiotic loaded spacers. However, these techniques show disadvantages such as breakage, bone cement abrasion and dislocation. Furthermore, prefabricated spacers are only available in limited sizes and specific antiinfective substances cannot be individually added [30, 31]. Some authors had developed techniques that use an interim prosthesis with a regular prosthesis stem and (thin) polyethylene cups for the two-stage surgery interval to overcome these disadvantages [4, 7]. We also used these techniques; however, because of acetabular complications in cases with severe bone loss, we modified this technique using a different acetabular fixation method [26]. We do not only use cement and a polyethylene inlay, but also a support ring, which prevents breakage of the cup and is still suitable for extensive acetabular bone loss. Minor differences to other techniques are the coating of the prosthesis with the patients’ own blood for easier removal and the individual mixture of antiinfective substances in the bone cement in each case.

The usage of temporary prosthetic implants means bringing in new avital metal and polyethylene surfaces into the joints that were considered septic prior to surgery. We were able to show, however, a reinfection-free rate of 92% with a very low spacer-related complication rate of 10% [26]. Data on a more favourable clinical outcome of patients treated with this technique are, however, still missing.

In septic revision surgery of hip arthroplasties, removing the original implant can be challenging. Two different surgical techniques are widely used. In the endofemoral approach, the femoral component is removed without osteotomy of the femur. Indications for this approach are loosened uncemented prostheses or cemented prostheses with easily removable cement. The more invasive method is the transfemoral approach, in which the femur is osteotomized to create a bone flap which allows for a better access to the proximal femur to mobilize implants or cement that are still firmly fixed in the diaphysis. After implant removal, the flap is placed into its original position again and usually fixed with a cerclage. The advantage of the transfemoral approach is the direct access to the proximal femur. The downside of this technique is, however, a significantly poorer clinical short-term outcome and a higher risk for a positive Trendelenburg sign [12, 16]. Data comparing endo- and transfemoral approaches are generally rare and, to the best knowledge of the authors, in the special case of septic two-stage surgery not available.

The aim of the present study was to evaluate the clinical results of this new spacer technique with an additional subgroup analysis of an endofemoral and transfemoral surgical technique. The clinical evaluation was designed as an explorative study. For the group comparison, we hypothesized that the transfemoral subgroup is inferior in terms of reinfection rate and clinical status during the interim phase due to the more invasive surgery.

## Material and methods

### Patients

86 patients with a septic two-stage revision operation with implantation of the custom-made spacer as previously described were included in the study [26]. The periprosthetic joint infection was diagnosed preoperatively in all cases according to the criteria of the Musculoskeletal Infection Society (MSIS) and the International Consensus on Musculoskeletal Infection (ICM) 2018 [27, 28].

### Surgical procedure

**Fig. 1:**
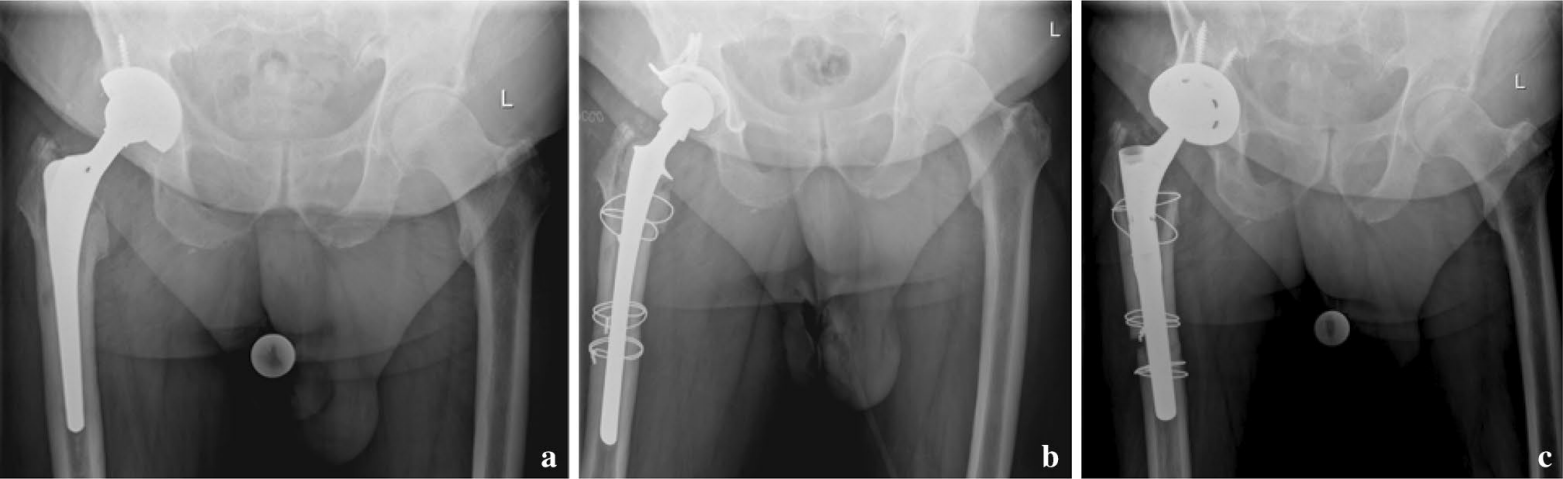
Transfemoral approach in case of a fully osteointegrated prosthetic stem. **a** Infected stem on the right side. **b** Transfemoral approach for the implantation of an interim prosthesis consisting of a cemented stem (Waldemar Link GmbH & Co. KG, Hamburg, Germany) and an acetabular Ganz-ring (fixed with only three screws) with a Müller flat-profile cup (ZimmerBiomet, Winterthur, Switzerland). The bony flap was refixed with 1.5 mm diameter cerclages. **c** Situation after second-stage surgery with the definitive prosthesis consisting of a TM-Cup Multihole and a Revitan stem (ZimmerBiomet, Winterthur, Switzerland)

45 cases were explanted endofemorally and 41 cases were explanted transfemorally. The transfemoral approach was performed with a modified Wagner technique as previously published [13, 15]. In brief, a posterolateral incision to the femur was applied and the posterolateral region of the femur was exposed via the septum intermusculare laterale with ligation of the perforating vessels. Following the preparation of the lateral circumference of the femur in the area where the estimated end of the osteotomy flap was going to be positioned, two 3.2 mm holes were drilled under cooling. One hole was positioned above the linea aspera and one hole was positioned 180 degrees ventromedial from the first hole. The osteotomy at the proximal ventromedial trochanter region was applied with a chisel at the vasto-gluteal border. In the next step the dorsolateral osteotomy was performed using a water-cooled oscillating saw, followed by the transverse osteotomy connecting the two drill holes. Then an osteotomy of the distal ventromedial at 3 cm was performed. The ventromedial osteotomy was completed in the proximal direction with a chisel driven blind under the vastus lateralis muscle to the proximal end of the osteotomy. The vastus lateralis muscle thus remains attached to the mobile bone flap. After implantation of the cement-covered interim stem, the bone flap was closed with two double cerclage wires (1.5 mm diameter) (Fig. 1). In the second-stage surgery, the bone flap was reopened and closed again with cerclages after the implantation of the new prosthetic components. All surgeries were performed in Orthopaedic Clinic Markgröningen.

Post-explantation a radical debridement followed and the custom-made interim prosthesis was implanted. The stem spacer component consisted of a regular cemented prosthesis stem. Prior to encasing the stem with bone cement supplemented with individually mixed antibiotics, it was coated with the patient’s own blood. Furthermore, implantation was performed with the cement being 6 min old to reduce the quality of interdigitation of cement. Both steps were carried out to facilitate later removal. The acetabular spacer consisted of a support ring [Müller cup, Ganz ring, or a Burch–Schneider-acetabular reinforcement ring depending on the acetabular bone defect (ZimmerBiomet, Winterthur, Switzerland)] and a polyethylene cup cemented herein. The support rings were fixed with two to maximal four screws and with an individual mixture of antibiotics in the cement according to the susceptibility of the microorganism.

**Fig. 2:**
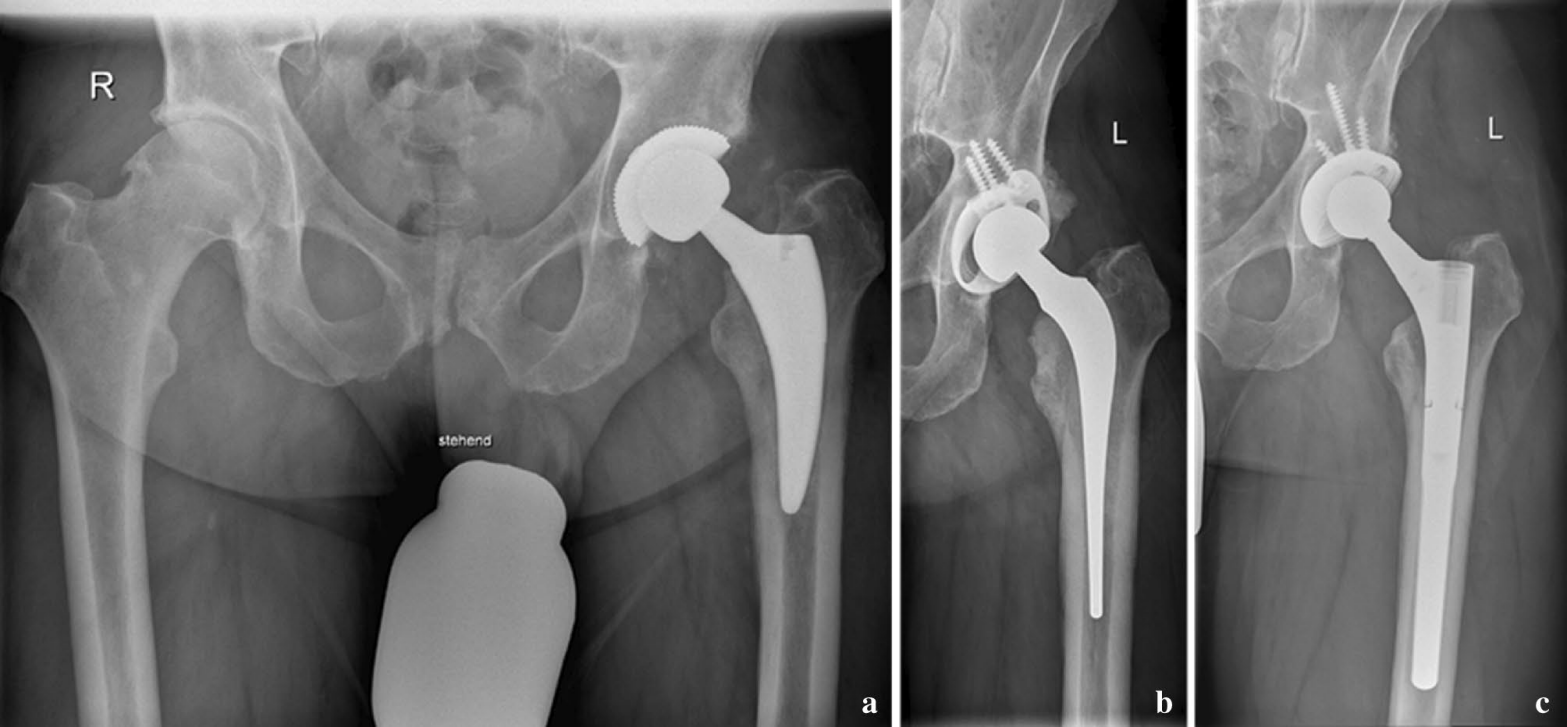
Endofemoral approach in case of a loosened cementless stem. **a** Infected Fitmore stem (ZimmerBiomet, Winterthur, Switzerland). **b** Implanted interim prosthesis consisting of a cemented stem and acetabular a Müller-ring with a Müller flat-profile cup (ZimmerBiomet, Winterthur, Switzerland). **c** Situation after second-stage surgery with the definitive prosthesis consisting of an Allofit S-cup and a Revitan stem (ZimmerBiomet, Winterthur, Switzerland)

**Fig. 3:**
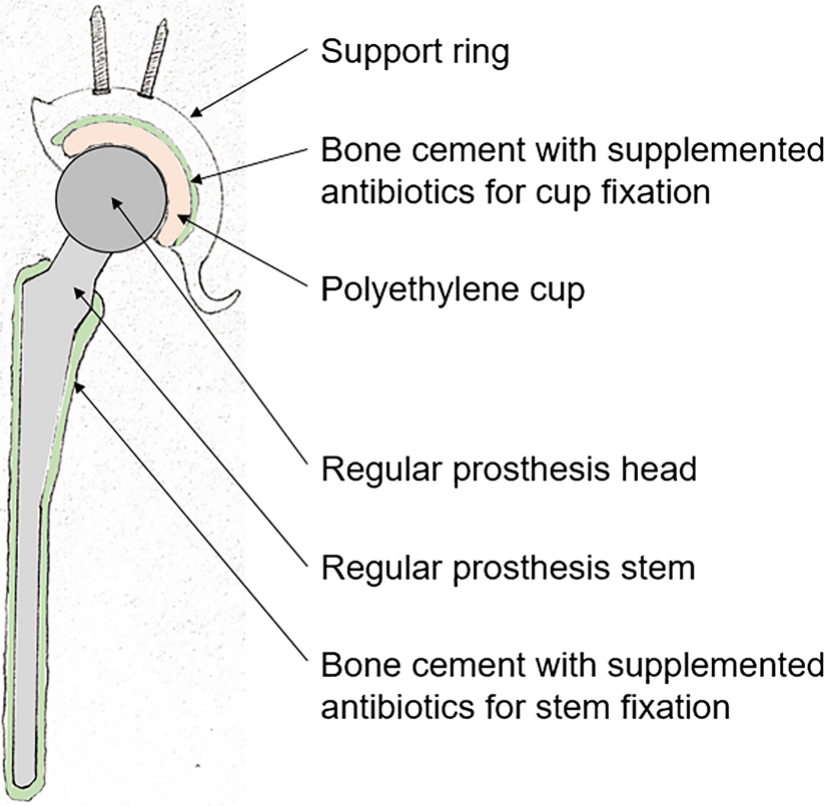
Schematic figure showing the spacer technique. Regular cemented prosthesis stem and polyethylene cup cemented in a support ring. The bone cement is supplemented with individually mixed antibiotics and it is already 6 min old when used to reduce interdigitation. Furthermore, the prosthesis components are coated with the patient´s blood to facilitate later removal of the implant from the cement

The femur and acetabulum component were articulated with a metal head on the polyethylene cup (Figs. 2, 3).

### Applied bone cement and administered antibiotic substances

Copal cement (Heraeus, Darmstadt, Germany) was used as industrially prepared cement. An individual and specific mixture of antibiotic substances was applied to the bone cement according to the antibiotic susceptibility profile of the microorganisms detected preoperatively. A maximum of 10% of the total cement powder weight was added as antibiotic substance to avoid mechanical problems. The cement of the spacer contained two antibiotic substances in 37 cases, three in 45 cases, and four in 3 cases (Table 1).

**Table 1:** Spacer cement and individual mixture of added antibiotic substances

Spacer cement	Individually added antiifective substances	Number
Copal^a^ G + C (gentamicin + clindamycin)		17
Copal G + C (gentamicin + clindamycin)	Vancomycin	43
Copal G + V (gentamicin + vancomycin)		20
Copal G + C (gentamicin + clindamycin)	Meropenem	2
Copal G + C (gentamicin + clindamycin)	Vancomycin, meropenem	4

^a^Heraeus, Darmstadt, Germany

### Postoperative regime

The individual parenteral antibiotic treatment started perioperatively. After 2 weeks of parenteral antibiotic therapy, the antibiotic treatment was changed to oral administration. The high bioavailability of rifampicin and ciprofloxacin allowed their oral administration already from the second day following surgery.

After 2 weeks of antibiotic treatment and mobilization with partial weight bearing on the operated leg, the patients were discharged from hospital. There was no restriction in range of motion for the operated joint except movements hazardous for dislocation.

During the re-implantation procedure, at least five biopsies were taken for bacteriological examination. Antibiotic treatment followed the same protocol as after the first-stage surgery.

After re-implantation of the new prosthesis, mobilization with partial weight bearing of 10 kg for a period of 6 weeks was allowed. Full weight bearing was gradually reached 3 months postoperatively as previously described by other authors for other cementless revision stems [13–17].

### Follow-up

Examination of all patients took place before the operation, 3, 6, 9, 12, 18, and 24 months postoperatively. Later follow-up examinations were based on a regular interval of every 2 years. Patients who no longer attended regular follow-up examinations, as described, were classified as lost to follow-up. Patients were judged definitely infection free according to Diaz-Ledezma et al. [9] if he or she was free from mortality related to periprosthetic joint infection, free from subsequent surgical intervention for periprosthetic joint infection, and if there was a microbiological and clinical absence of the infection for at least 2 years. If there was suspicion of a periprosthetic joint infection at a follow-up, the diagnosis was again ruled out or confirmed with the MSIS criteria 2014 the ICM criteria 2018.

### Clinical score

The Staffelstein-Score was used to record the clinical and functional results. This is a score validated in German to record objective and subjective criteria for hip arthroplasty. It strongly resembles the Harris Hip Score [21], which does not exist in a validated form in German for which reason the Staffelstein-Score was applied. Items which are assessed similarly in both scores are “Pain”, “Support”, “Walking distance”, “Limp”, “Activities” (shoes, socks), “Stairs”, “Public transportation” and “Range of motion”.

### Statistical analyses

Statistical analyses were conducted using IBM SPSS Version 20 (IBM, Armonk, NY, USA) and Microsoft Excel (Microsoft, Redmond, WA, USA). Distributions of variables within the groups were assessed by histograms and a non-parametric approach was chosen. Continuous variables are presented as medians and ranges, and categorical variables as frequencies. Comparison between groups was performed by Mann–Whitney *U* test, Wilcoxon test or Chi-square test as appropriate. All reported *p* values are two-sided, with an alpha level of 0.05 and have not been adjusted for multiple testing. Survival is presented with a Kaplan–Meier curve.

### Ethical approval

The study was conducted according to the guidelines of the Declaration of Helsinki and was approved by the local ethics board of the University Hospital of Tübingen (registration number 418/2021BO2).

## Results

### Patients

Septic two-stage prosthesis revision surgery was performed in 86 patients with late periprosthetic infection of the hip endoprosthesis between May 2013 and September 2017. 29 women and 57 men formed the study collective. Median age was 70 (27–92) years. The average body mass index was 29.3 ± 6.1 kg/m^2^. Diabetes mellitus and rheumatoid disease were known in 12 (11%) and 7 patients (6%), respectively. ASA classification was as follows: ASA 1: 2 patients, ASA 2: 38 patients, ASA 3: 45 patients and ASA 4: 1 patient. With 71%, the majority of explanted prostheses were primary implants (69% cementless, 18% hybrid, 10% cemented, 1.5% bipolar and 1.5% surface replacement prostheses). 29% of explanted prostheses were revision implants.

21 patients had already undergone one septic revision and three patients had already undergone multiple revision operations.

In 45 patients, an endofemoral approach and in 41 patients a transfemoral approach were performed. There was no statistical difference regarding sex (*p* = 0.175), body mass index (*p* = 0.935), diabetes mellitus (*p* = 0.244), rheumatoid diseases (*p* = 0.470), ASA classification (*p* = 0.890), type of explanted prosthesis (*p* = 0.161), previous septic revisions (*p* = 0.239), and follow-up (*p* = 0.384) between both groups. However, patients with endofemoral approach were of significantly younger age (*p* = 0.023) (Table 2).

**Table 2:** Group comparison between endofemoral and transfemoral approach

	Endofemoral	Transfemoral	*p* value
Number	45	41	
Median age (range) [years]	69 (27–92)	73 (43–85)	0.023
Sex (w:m in %)	27: 73	41: 59	0.175
Mean body mass index (SD) [kg/m^2^]	30 (± 7)	29 (± 5)	0.935
Diabetes mellitus (%)	5 (4)	9 (7)	0.244
Rheumatoid diseases	3 (2)	5 (4)	0.470
Previous septic revisions (%)	10 (22)	14 (34)	0.239
Median follow-up (range) [months]	51 (6–86)	47 (2–87)	0.394

### Microbiological etiology

Most frequently detected were *Staphylococcus epidermidis* and *Cutibacterium acnes* (Supplementary material, Table 1). In two cases, there was no causative microorganism detected. However, because of a fistula communicating with the joint and elevated CRP in one case and elevated CRP, a positive histopathological result and elevated polymorphonuclear neutrophil percentage in the joint fluid in the other case, they both met the criteria for a PJI. In 17 cases two different and in 1 case three different causative organisms were identified. In one case, even four microorganisms were found positive.

### Follow-up

Median follow-up until dropout was 50 (2–87) months. Dropout reasons were reinfection (8 cases), death (6 cases), end of follow-up (53 cases) or lost to follow-up (19 cases). The 19 patients were lost to follow-up at 2–55 months (median: 24 months) postoperatively. No perioperative deaths were observed in the collective. The reported dropouts due to death were not directly linked to septicemia or treatment-specific complications associated with the infected prosthesis.

**Fig. 4:**
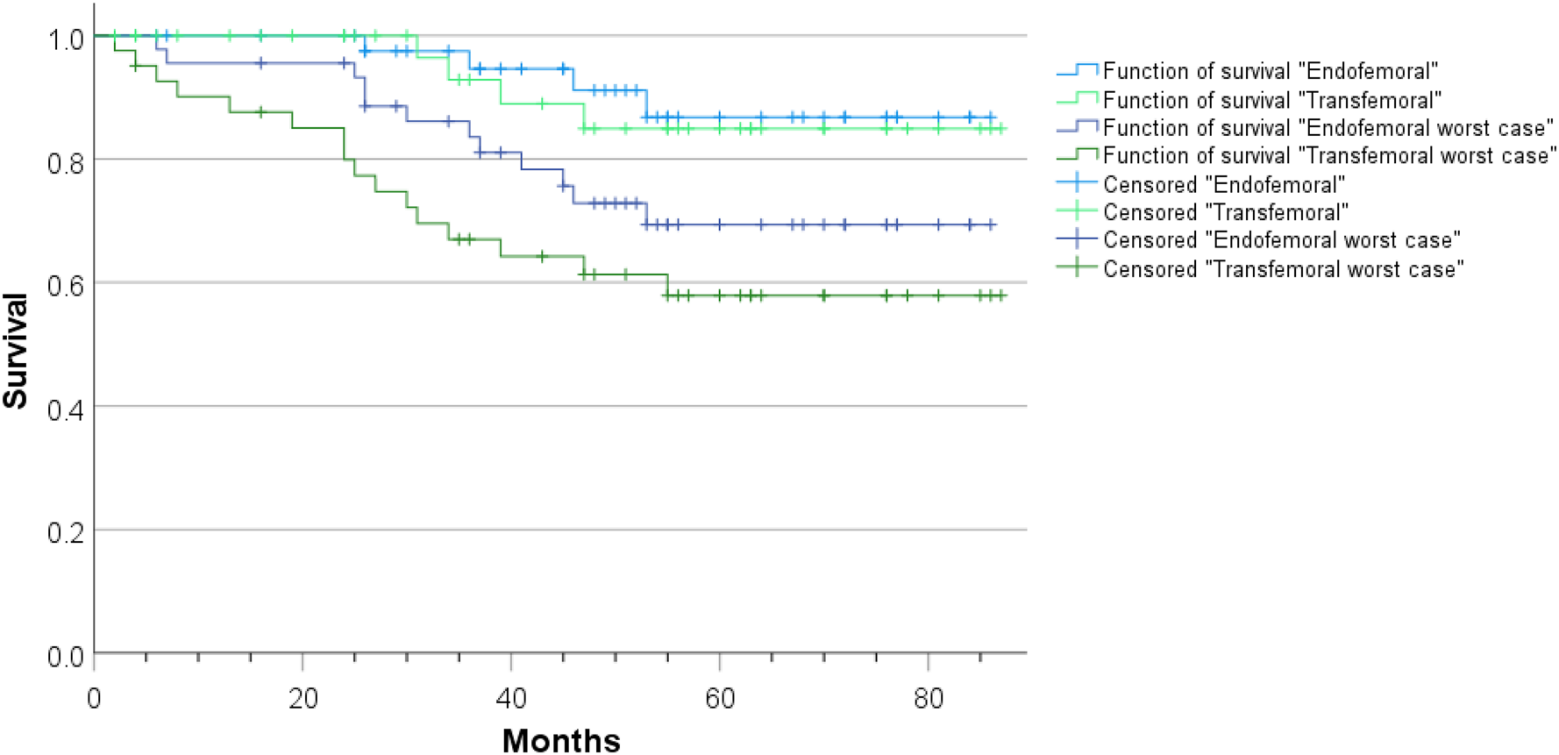
Kaplan–Meier curve for the cumulative survival after two-step septic revision (light blue: endofemoral, *n* = 45; light green: transfemoral, *n* = 41). Reasons for censoring were death (6 cases, not directly linked to septicemia or treatment-specific complications associated with the infected prosthesis) lost (19 cases) or end of follow-up (53 cases). Additionally, a worst case scenario calculation defining lost-to-follow-up as failure is included (dark blue: endofemoral, *n* = 45; dark green: transfemoral, *n* = 41). Failure was defined as reinfection

At the end of the follow-up, 78 cases out of the whole collective (90%) had no reinfection and 8 cases (10%) had to be classified as “reinfected”. Of the eight cases with reinfection, four were in the “endofemoral” (10%) and four in the “transfemoral” (12%) group. This distribution was not statistically different (*p* = 0.590). We additionally performed a worst case survival analysis, classifying the lost to follow-up cases as reinfected. Considering the worst case scenario, the reinfection rate was 33%. Survivorship (reinfection-free) at 36 months postoperatively was 94.6% (95% CI 91.6–100%) for the endofemoral group and 92.9% (95% CI 83.8–100%) for the transfemoral group. The difference for the function of survival between the groups was not statistically significant (log-rank test, *p* = 0.742). Calculating the worst case scenario, the survivorship at 36 months postoperatively was 83.6% (95% CI 73.1–95.5%) and 67.0% (95% CI 53.8–83.4%) for the endofemoral and the transfemoral collective, respectively (Fig. 4). However, the difference in the function of survival is also not statistically significant in the worst case scenario (log rank test, *p* = 0.184).

In three cases (7%: one case with *Staphylococcus epidermidis*, two cases with *Staphylococcus capitis*) in the endofemoral collective and in one case (3%: *Staphylococcus epidermidis*) in the transfemoral collective the samples taken during the reimplantation operation were positive for bacterial infection. However, in all three cases there was only just one positive culture out of at least five samples. Therefore, these samples were classified as contamination. None of these cases with a positive detection of microorganisms at the time of the reimplantation operation had a reinfection during follow-up.

### Clinical status during the interim phase

**Fig. 5:**
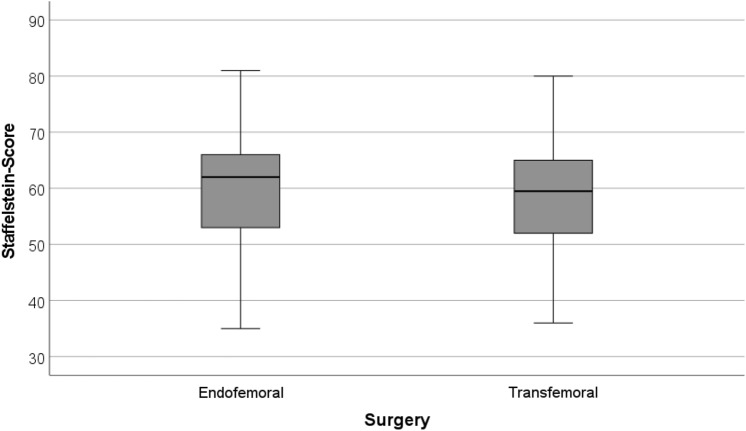
Box plots for the Staffelstein-Score (“Endofemoral”: endofemoral surgery; “Transfemoral”: transfemoral surgery)

The whole collective presented a median Staffelstein-Score with the spacer at the end of the interim phase of 60 (range 31–81) out of 120 possible maximum points. Comparing the clinical status of both procedures, with a median of 62 (range 31–81) for the endofemoral and of 60 (range 32–80) for transfemoral surgery, there was no statistically significant difference (*p* = 0.410) (Fig. 5).

### Complications

Spacer-related complications were recorded in seven cases (8%) of the total collective with the most frequent complication being articular dislocation in five cases (7%). Furthermore, two cup breaking out were observed (2%). Revision operation of the spacer for spacer complication was necessary in five cases (7%). Comparing the endofemoral and transfemoral surgery regarding complications, there was no statistically significant difference with three cases in each group (*p* = 0.704).

## Discussion

We recently reported about a spacer technique for septic two-stage revision surgery in PJI of hip arthroplasties [26]. In the present study, we evaluated the clinical status of this spacer technique and compared the subgroup of the endofemoral and transfemoral groups. The clinical status could be classified as moderate. Furthermore, we had to reject our hypothesis that the transfemoral group was inferior regarding reinfection rate and clinical status during the interim phase.

In general, the implantation of mobile, articulating spacers in two-stage revision has the advantage of easier reimplantation and helps to maintain the patients’ mobility [5]. Techniques without spacer placements do not pose the risk of spacer complications. However, disadvantages are the inferior functional outcome [25] and the lack of release of local antiinfective substances when not using other antiinfective agent delivering materials (e.g., antibiotic-loaded sponges or gels). This may lead to lower rates of bacterial eradication [32]. Our presented technique combines the advantages of a single-stage procedure with maintaining mobility and stability of the joint while still allowing the radical debridement of two-stage procedures with the intermitting targeted antiinfective therapy. The implantation of stable interim implants with a tribologically good articulation prevents cement particle abrasion and has additionally the advantage that an antiinfective substance can be added to the cement to individually address the causative microbiological spectrum.

With a reinfection-free rate of 92% [26], this technique appears equal or possibly superior compared to collectives with Girdlestone situations, handmade antibiotic-impregnated articulating polymethyl methacrylate spacers or large mixed technique collectives [1, 6, 22, 26, 33]. The risk of implanting avital metal and polyethylene prostheses parts in the interim phase seems to have no microbiological disadvantage when embedded in the overall therapeutic regime. In our present study, reimplantation microbiology was positive in 7% of all cases at the end of the interim phase. These results are comparable with or even lower than other studies investigating microbiological findings at reimplantation ranging from 5 to 14% positive samples at reimplantation [3, 26, 29]. Having only one positive out of five taken samples in cases with positive reimplantation microbiology, all these cases were classified as contaminated.

With 8% spacer-related complications, this rate was very low when compared with other studies reporting rates of 26% or even higher [2, 10, 23].

With respect to the functional status of the patients in the interim phase, Staffelstein-Score values were of a median score of 60 (range 31–81). These results can be classified as moderate. They need, however, to be considered in the context that only partial weight bearing was allowed during that time. Unfortunately, there is only one comparable work in the literature regarding clinical evaluations of interim spacers. With a Harris Hip score of 38 points (maximum 100 points), the result is also at best moderate [22]. However, a comparison is hardly possible since in that study several spacer techniques were analyzed together. Knowing that our patients were allowed a free range of motion and 10–20 kg partial weight bearing compared with toe touch weight bearing or impaired range of motion, our patients might have a clinical advantage [6]. Our results imply that patients with our technique are moderately impaired in daily life, but are still able to fulfill their activities of daily living independently.

Another aspect of two-stage revision surgery in hip arthroplasty is the decision between an endo- and transfemoral procedure. Interestingly, in our collective there was no statistical difference regarding reinfection rate between both procedures (*p* = 0.590). Previous studies had reported poorer clinical short-term outcome of transfemoral procedures [12, 16]. With a median Staffelstein-Score around 60, the endo- and transfemoral procedure did, however, not clinically differ significantly (*p* = 0.410). Complication rates were also comparable between both procedures. In our opinion, a transfemoral procedure appears to be a good alternative whenever an endofemoral procedure is not feasible. What has to be kept in mind is that we only analyzed the clinical status in the interim phase. The clinical outcome after reimplantation can differ and previous studies imply that the transfemoral procedure needs a longer time for rehabilitation [16].

One strength of this study is a good comparison between an endo- and transfemoral procedure at the end of the interim phase, as there was a consistent pre- and postoperative treatment regime for both procedures. We were able to provide clinical data of our procedure with custom-made interim spacers. However, since the decision for the approach was based on clinical indications, the assignment to the groups was not randomized. This limits the comparability. Due to the retrospective analysis of the study, we were also not able to provide the subscores for pain, activities of daily living and range of motion, which may be further subspecified in future studies. Calculating the reinfection rate and the reinfection-free survivorship, 19 cases were lost to follow-up. Although all 19 cases seemed to be reinfection-free at their last follow-up, they possibly might have developed a reinfection over time. For this reason, we additionally calculated a worst case scenario survivorship, which would thus represent the worst hypothetically possible outcome.

## Conclusions

In this study, we were able to provide clinical data of patients with a new type of interim spacers. Our procedure with implanting prostheses parts in the interim phase shows promising clinical results. Furthermore, using this technique, we saw no clinical or infectiological differences between patients that had undergone an endofemoral or a transfemoral approach.

## Supplementary Information

Below is the link to the electronic supplementary material.

Supplementary file1 (DOCX 15 KB)

## Data Availability

The data that support the findings of this study are available from the corresponding author, MM, upon reasonable request.
