# Supplementary material for: Clinical evaluation of a new technique for custom-made spacers in septic two-stage revision of total hip arthroplasties
Source: Arch Orthop Trauma Surg. 2023 Jan 5;143(8):5395–403. doi: 10.1007/s00402-022-04748-z (PMC10374709; doi:10.1007/s00402-022-04748-z)
Supplement: Supplementary file 1 — Supplementary file1 (DOCX 15 KB) [file 402_2022_4748_MOESM1_ESM.docx]

**Table 1.** Identified microorganisms and the number of detections

| **Classification** | **Microorganism** | **Number** | **% of cases infected by this pathogen** |
| --- | --- | --- | --- |
| **Gram-positive cocci** | *Staphylococcus epidermidis* | 30 | 35 |
|  | *Staphylococcus aureus* | 9 | 10 |
|  | *Enterococcus faecalis* | 5 | 6 |
|  | *Staphylococcus hominis* | 5 | 6 |
|  | *Staphylococcus capitis* | 4 | 5 |
|  | *Staphylococcus warneri* | 2 | 2 |
|  | *Staphylococcus caprae* | 1 | 1 |
|  | *Staphylococcus lugdunensis* | 1 | 1 |
|  | *Staphylococcus haemolyticus* | 1 | 1 |
|  | *Staphylococcus saccharolyticus* | 1 | 1 |
|  | *Staphylococcus saprophyticus* | 1 | 1 |
|  | *Streptococcus sanguinis* | 1 | 1 |
|  | *Streptococcus salivarius* | 1 | 1 |
|  | *Streptococcus agalactiae* | 1 | 1 |
|  | *Streptococcus anginosus* | 1 | 1 |
|  | *Granulicatella adiacens* | 1 | 1 |
| **Gram-positive rods** | *Cutibacterium acnes* | 27 | 35 |
|  | *Cutibacterium granulosum* | 4 | 5 |
|  | *Listeria monocytogenes* | 3 | 3 |
| **Gram-negative rods** | *Escherichia coli* | 3 | 3 |
|  | *Klebsiella pneumoniae* | 1 | 1 |
|  | *Bacteroides fragilis* | 1 | 1 |
|  | *Proteus mirabilis* | 1 | 1 |
|  | *Morganella morganii* | 1 | 1 |
